# A German climbing study on depression: a bouldering psychotherapeutic group intervention in outpatients compared with state-of-the-art cognitive behavioural group therapy and physical activation – study protocol for a multicentre randomised controlled trial

**DOI:** 10.1186/s12888-019-2140-5

**Published:** 2019-05-17

**Authors:** Lisa Dorscht, Nina Karg, Stephanie Book, Elmar Graessel, Johannes Kornhuber, Katharina Luttenberger

**Affiliations:** 1 Department of Psychiatry and Psychotherapy, Centre for Health Services Research in Medicine, University Hospital Erlangen, Friedrich-Alexander-Universität Erlangen-Nürnberg (FAU), Schwabachanlage 6, 91054 Erlangen, Germany; 2 Department of Psychiatry and Psychotherapy, University Hospital Erlangen, Friedrich-Alexander-Universität Erlangen-Nürnberg (FAU), Schwabachanlage 6, 91054 Erlangen, Germany

**Keywords:** Depression, Exercise program, Physical activity, Sports, Bouldering, Rock climbing, Psychotherapy

## Abstract

**Background:**

Besides classical approaches for treating depression, physical activity has been demonstrated to be an effective option. Bouldering psychotherapy (BPT) combines psychotherapeutic interventions with action-oriented elements from the field of climbing. The aim of this study is to investigate the effectiveness of BPT compared with a home-based exercise program (EP - active control group, superiority trial) and state-of-the-art cognitive behavioural therapy (CBT – non-inferiority trial).

**Methods:**

The study is being conducted as a multicentre randomised controlled intervention trial at three locations in Germany. Participants are being randomised into three groups: BPT, CBT, or EP, each with a 10-week treatment phase. A power analysis indicated that about 240 people should initially be included. The primary outcome of the study is the Montgomery and Asberg Depression Rating Scale (MADRS) directly after the intervention. Additional measurement points are located three, six, and 12 months after the end of the intervention. The data are being collected via computer-assisted telephone interviews. Statistical analyses comprise regression analyses to test for the superiority of BPT over EP. To test for the non-inferiority of BPT and CBT, a non-inferiority margin of 1.9 points in the Patient Health Questionnaire (PHQ-9) and two non-inferiority margins for the MADRS (half of the two smallest Cohen’s d values from the current meta-analyses) was predefined. The mean difference between CBT and EP is being used as a supplementary equivalence margin.

**Discussion:**

This is the first study to investigate the effect of a bouldering psychotherapy (BPT) on outpatients’ depressive symptoms compared with mere physical activity (superiority analysis) and state-of-the-art cognitive behavioural therapy (CBT, non-inferiority analysis).

Methodological strengths of the study are the elaborated, multicentred, randomised, controlled design. Assessors are blinded with regard to group allocation which leads to high objectivity. The study is conducted in a naturalistic setting, which leads to high external validity. Methodological limitations might be the clinical heterogeneity of the sample, which may dilute the intervention effects.

**Trial registration:**

ISRCTN12457760 (Registration date: 26 July 2017, retrospectively registered).

## Background

With a lifetime prevalence of 12.5 [1] and a 12-month prevalence of 3.2% [2], depression is one of the most common diseases worldwide. Estimations of the WHO indicate that by 2020, depression will be the second leading cause of health impairment (after cardiovascular diseases), and by 2030, it will be the most frequent impairment in industrialised nations [3, 4]. In line with clinical guidelines for the treatment of depression, medication and psychotherapy are the approaches that are most commonly recommended [5, 6]. The gold standard in the psychotherapeutic treatment of depression is cognitive behavioural therapy. With effect sizes (Hedges’ *g*) ranging from 0.55 to 0.98 (moderate to large effects) in metaanalyses [7] it has shown to be at least as effective as antidepressants [5, 8]. Especially regarding psychotherapy, there is a vast gap between supply and demand owing to a lack of therapeutic resources. In the UK, only 10% of the people suffering from anxiety or depression receive psychological treatment [9]. Another challenge is that it is difficult to engage and retain patients in psychological care. Only about 52.2% of depressive individuals living in Germany actively seek professional help [10]. Also in the UK, only half of the patients who receive psychological treatment from a general practitioner attend two or more sessions [11]. These findings highlight the need for new therapies that are as effective as established treatment options and at the same time easier for patients to access, more attractive, and less stigmatized. Besides the classical approaches of psychotherapy and medication, physical activity has also been demonstrated to be an effective treatment option [12–15]. On the basis of effect sizes (Cohen’s *d*) that have been reported to lie between 0.62 and 0.82 (moderate to strong effects), physical activity has been added to the German guidelines for the treatment of depression as a supplementary therapeutic method [5].

One type of physical activity that has become increasingly popular in recent years and is spreading all over the world is climbing, notably bouldering. Bouldering means climbing without a rope on rocks or artificial indoor climbing walls at a height that permits the climber to jump off. Climbing halls provide climbing routes with different levels of difficulty (coded with different colours), enabling persons with varying levels of physical fitness to boulder together without feeling over- or unchallenged. Climbing and bouldering have gained increased attention in clinical practice and are nowadays often used as part of the overall treatment plan for health problems [16, 17]. Earlier research showed that therapeutic climbing enables the stimulation of cognitive, emotional, and physical development as well as learning processes [18]. Furthermore, it can offer a person an opportunity to become more active and can be a learning opportunity in which a person can set realistic goals. Finally, mastering “bouldering problems” has been found to have a positive impact on a climber’s self-esteem [19]. Owing to the different levels of difficulty, even beginners can experience a sense of achievement, which sets up positive reinforcement processes that might be especially helpful in the treatment of depression [18].

Existing studies on the therapeutic effects of climbing and bouldering have suggested that climbing/bouldering has positive effects on health issues such as chronic pain [20–22], multiple sclerosis [23], cerebral palsy [24], and severe haemophilia A [25] as well as on psychological problems such as ADHD [19, 26], anxiety disorders [19], and eating disorders [19]. Such effects might be moderated by an increase in cognitive functioning [16, 19], self-esteem, self-confidence, self-efficacy [19, 27], and social competences [16, 19]. Unfortunately, existing studies have suffered from various methodological problems: Some studies were case studies or descriptive reports with low case numbers [16, 19, 28]; some used only self-developed, unvalidated questionnaires or unstandardised interviews [29]; most did not randomly assign participants to intervention or control groups [30], and some used no control group at all [31]. Thus, a systematic review of existing randomised controlled studies on climbing/bouldering for preventing and treating health problems found only very low-quality evidence of improvements through therapeutic climbing and led to the conclusion that future studies should have a sufficient sample size, should use patient important outcomes, should be registered prospectively, and finally, should be reported in accordance with the CONSORT statement [31].

With respect to treating depression, some case reports and observational studies have reported positive effects of climbing and bouldering on depressive symptoms [16, 19, 30, 32]. Recently, a controlled – but not randomised – trial including 40 in-patients suffering from major depressive disorder showed the impact of rock climbing on acute emotion regulation strategies [17]. Positive affect and coping emotions significantly increased, while negative affect and depressiveness significantly decreased directly after the climbing session in comparison with a relaxation control group. Between 2013 and 2015, our work group conducted a randomised controlled pilot study to investigate the effect of a bouldering intervention on depressive symptoms. A total of 94 patients were randomly assigned to either a waitlist control group or an intervention group, which included eight sessions of bouldering psychotherapy. Different health outcomes were assessed with standardised psychological self-report questionnaires before and after the eight-week intervention period as well as 16 weeks after the end of the therapy. Participants in the intervention group reported a significant reduction in depressive symptoms compared with the control group, even when other types of physical activity was controlled for [33, 34]. With an effect size (Cohen’s *d*) of 0.77 (moderate effect) the effect of the bouldering intervention on depressive symptoms was comparable to other short-term group therapies [35] and to physical activity (see above).

## Methods

### Aims and hypothesis

The aim of the current study, StudyKuS (Studie**KuS** – **K**lettern **u**nd **S**timmung; engl. Climbing and Mood), is to investigate a) whether our newly developed bouldering psychotherapy is more effective in reducing depressive symptoms than physical activity alone and b) whether the effect of our therapy is comparable to current state-of-the-art group psychotherapy. For this purpose, we are comparing our bouldering psychotherapeutic intervention (BPT) with a state-of-the-art cognitive behavioural group therapy (CBT) and a home-based exercise program (EP - active control group) using a randomised and controlled but nevertheless naturalistic design.

#### Research hypothesis

I. Bouldering psychotherapy (BPT) leads to a significantly greater reduction in depressive symptoms in outpatients with depression than mere physical activity in the form of a home-based exercise program (EP).
II. The positive effect of bouldering psychotherapy (BPT) on reducing depressive symptoms in outpatients with depression is not inferior to the effect of cognitive-behavioural psychotherapy (CBT).

In this article, we describe the StudyKuS study protocol, which serves as a reference for forthcoming papers that report the results of the study.

### Study design and setting

**Fig. 1:**
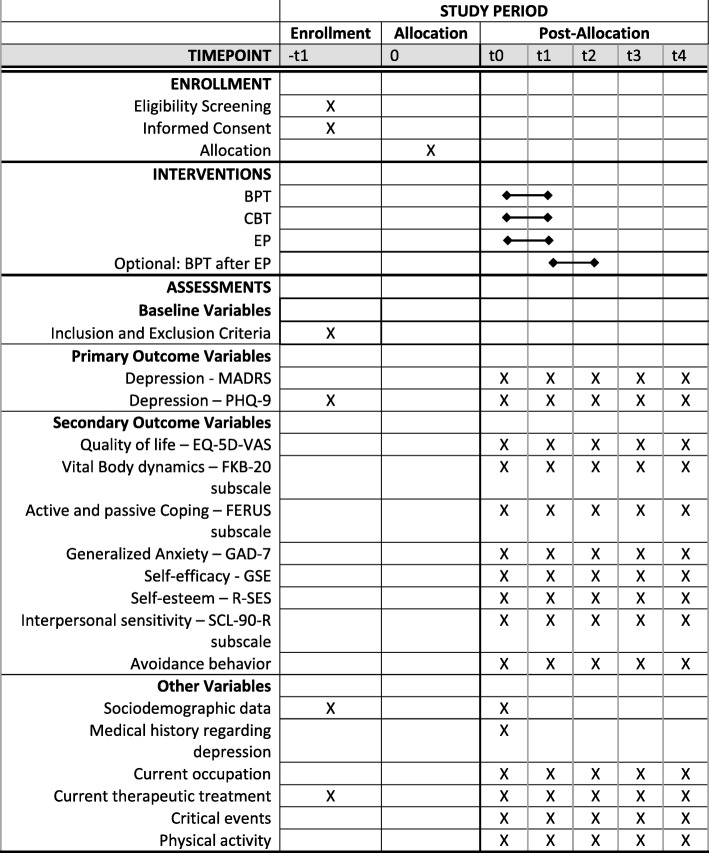
Participant timeline. Abbreviations: BPT: bouldering psychotherapy; CBT: cognitive behavioural therapy; EP: exercise program; EQ-5D: EuroQol Five Dimension Questionnaire; FERUS: Questionnaire on Resources and Self-Management Skills; FKB-20: Body Image Questionnaire; GAD-7: Generalized Anxiety Disorder 7; GSE: General Self-Efficacy Scale; MADRS: Montgomery-Asberg Depression Rating Scale; PHQ-9: 9-Item Patient Health Questionnaire; R-SES: Rosenberg Self-Esteem Scale; SCL-90: Symptom-Checklist; SD: Standard Deviation; VAS: visual analogue scale.

To test the research hypotheses, a randomised, controlled, multicentre, prospective longitudinal study with three arms is being conducted. Recruiting for the StudyKuS began in 2017 in three different regions in Germany: a) the Erlangen/Nuremberg/Fuerth region (metropolitan region of three cities), b) the Weyarn region (rural area surrounding Munich), and c) the Berlin (capital) region. Each intervention is being carried out by two therapists (with the exception of the EP, which is supervised by the study’s headquarters) over a period of 10 weeks in groups with a maximum of 11 participants. All of the three interventions in one region are taking place during the same time period and are being conducted in consecutive waves – with four waves in the Erlangen/Nuremberg/Fuerth region as well as the Weyarn region and two waves in the Berlin region. Participants in one region and one wave are randomly allocated to one of the three groups (BPT, CBT or EP). Data are being collected via computer-assisted telephone interviews (CATIs) before and directly after the intervention as well as three, six, and 12 months after the treatment. A data monitoring and safety board (DMSB) is established (EG and JK) and supervises study conduction and severe adverse events (SAEs). All procedures have been approved by the Friedrich-Alexander-Universität Erlangen-Nürnberg Ethics Committee (Ref. 360_16 B). The participant timeline is presented in Fig. 1. Trial Registration Data are presented in Table 1 (ISRCTN12457760, Registration date: 26 July 2017, registered retrospectively).

**Table 1:** Trial Registration Data

Data category	Information
1. Primary registry and trial identification number	ISRCTN12457760
2. Date of registration in primary registry	26 July 2017
3. Secondary identifying numbers	–
4. Source(s) of monetary or material support	OH-DO-KWAN Stiftung Ludmilla Pankofer und Carl Wiedmeier Förderverein Kletterzentrum Aufwärts in Miesbach e.V.
5. Primary sponsor	OH-DO-KWAN Stiftung Ludmilla Pankofer und Carl Wiedmeier
6. Secondary sponsor(s)	Förderverein Kletterzentrum Aufwärts in Miesbach e.V.
7. Contact for public queries	see Point 8
8. Contact for scientific queries	PD Dr. Katharina Luttenberger, katharina.luttenberger@uk-erlangen.de
9. Public title	Study KuS (Klettern und Stimmung - Climbing and Mood), a combination of bouldering and psychotherapy for treating depression
10. Scientific title	Study KuS (Klettern und Stimmung - Climbing and Mood) - Prospective investigation of the effectiveness of a combination of bouldering and psychotherapy in comparison with an active control group and cognitive behavioural group therapy for patients suffering from depression in an outpatient setting
11. Countries of recruitment	Germany
12. Health condition(s) or problem(s) studied	Depression
13. Intervention(s)	Study arm 1: Intervention group receiving the combination of bouldering and psychotherapy
	Study arm 2: Intervention group receiving cognitive-behavioural therapy
	Study arm 3: Active control group
1.14. Key inclusion and exclusion criteria	Ages eligible for study: adults; Sexes eligible for study: both
	Inclusion criteria: 1. Depression, measured by the patient’s score on the PHQ-9 (Cut-off ≥8) 2. Informed consent to participate in the study (especially regarding randomised allocation and data acquisition) 3. Ability to come to the therapy locations
	Exclusion criteria: 1. Acute suicidality 2. Severe psychiatric disorder (psychosis, mania, substance abuse) 3. Physical contraindication for climbing due to disorder or pregnancy 4. BMI < 17.5 or > 40 5. Age < 18 years 6. Actual participation in group psychotherapy 8. Began using psychiatric medication within the last 8 weeks 9. Began psychotherapy within the last 8 weeks 10. Planned inpatient stay during therapy
15. Study type	Randomised controlled intervention study
16. Date of first enrollment	June 2017
17. Target sample size	199
18. Recruitment status	complete
19. Primary outcome(s)	Depression measured using the score on an observer rating scale (Montgomery Asberg Rating Scale, MADRS) by computer-assisted telephone interviews (CATI)
20. Key secondary outcomes	e.g., body image, anxiety, social phobia, self-esteem, coping

### Sample size estimation

A power analysis was computed on the basis of the authors’ previous experience with the BPT in the pilot study in which a Cohen’s d of 0.77 was reached [33]. To address Hypothesis I, a slightly lower effect size of 0.6 was assumed. Thus,61 patients will be needed in the BPT group and 61 patients in the EP group (alpha = .05, power = .9).

To address Hypothesis II, a non-inferiority margin of 1.9 points (SD = 3.8) on the PHQ-9 (as proposed in the literature [36]) was used, which resulted in a total of 69 patients in the BPT group and 69 patients in the CBT group (alpha = .05, power = .9). Thus, there should be 61 patients in the EP group, 69 patients in the BPT group, and 69 patients in the CBT group, for a total of 199 patients. To account for dropouts between t0 and t1, 20% more patients are added (based on the drop-out-rate of our pilot study), resulting in 40 additional patients for a total of 239.

Therefore, 10 waves in three therapy centres (two waves in Berlin, four waves in Weyarn, four waves in Erlangen) were planned. Each wave was designed to include 24 participants on average, of which 20 were expected to complete the respective intervention.

Also from the data of the pilot study, in which similar recruitment modalities were applied, a failure of approx. 40% of patients who do not meet the inclusion criteria must be expected. This means that a total of approximately 400 patients should be screened.

### Recruitment strategies

Subjects are being recruited in several ways: Informational material (e.g. flyer, posters) is being distributed throughout the three study locations at psychiatric hospitals, locally based psychotherapist offices, primary care physician offices, pharmacies, and other psychological services (e.g. support groups). Information is also being sent to psychotherapists, psychiatrists, and primary care physicians requesting them to share it with their patients. Prior to each interventional wave, press releases are being issued and addressed to different local newspapers and radio stations, and presentations are being given at local events (e.g. “Day of Action against Depression”). On the web, a homepage (http://www.psychiatrie.uk-erlangen.de/med-psychologie-soziologie/klettern-und-stimmung-die-studie-kus/) and a Facebook account were created and are being updated with current information regarding the study on a regular basis.

### Eligibility of participants

All individuals interested in the study are being invited to attend information sessions held by the study personnel, where they are provided with all relevant information about the conditions surrounding their participation in the study (e.g. randomisation). People who are willing to participate are asked to fill out a short screening questionnaire (scanning for inclusion and exclusion criteria) and to provide their written informed consent.

#### Inclusion and exclusion criteria

To increase external validity, only a few inclusion and exclusion criteria are being applied. *Inclusion criteria* consist of acute depressive symptoms, informed consent to participate in the study (especially regarding randomised allocation and data acquisition), and the ability to reach the therapy locations. The presence (or absence) of depression is operationalised as a PHQ-9 score of at least 8 points, ensuring a high level of sensitivity to all depressive disorders [37]. *Exclusion criteria* are age younger than under 18 years, a Body Mass index (BMI) under 17.5 or over 40, contemporary participation in another psychotherapeutic group therapy, started taking psychiatric medication within the last 8 weeks (medication started before 8 weeks is not a reason for exclusion), started individual psychotherapy within the last 8 weeks (same as for medication), a planned inpatient stay during the intervention period, physical contraindication for bouldering (physical disorders or pregnancy), specific psychiatric disorders (psychosis within the last 5 years, a manic episode within the last 5 years, substance addiction with substance abuse within the last year, Borderline diagnosis with self-harming behaviour during the last year), and acute suicidality. If it turns out that someone is in a suicidal crisis, appropriate steps following good clinical practice and a suicide risk management plan should be taken by the study personnel, if necessary by arranging for inpatient admission. For safety reasons, all participants are obliged to sign an anti-suicide contract for the duration of the study. All inclusion and exclusion criteria are assessed via self-reports with the screening questionnaire. In cases of unclear fulfilment of the inclusion or exclusion criteria (especially regarding relevant psychiatric diagnoses), potential participants are personally interviewed by the study personnel.

### Randomisation

All individuals meeting the inclusion criteria in one study region are being randomised into one of the three groups (BPT, CBT, EP). Randomisation is stratified by sex and severity of depression according to the PHQ-9 score from the screening questionnaire (9–14 mild, 15–19 moderate, 20–27 severe depression). After randomisation, participants are informed about their allocation and provided with all necessary information about group participation. Randomisation is performed with a computer-based system by the Institute of Medical Informatics, Biometrics, and Epidemiology (IMBE) of the Friedrich-Alexander-Universität Erlangen-Nürnberg.

#### Masking

Participants know which treatment condition they are in, but the interviewers who conduct the CATIs and thus assess the outcomes of the study are blinded to participants’ allocations. Prior to each interview, all participants are reminded of the confidential nature of their allocation and asked not to tell the interviewer which treatment they receive.

### Interventions

#### Bouldering psychotherapy (BPT)

Bouldering is defined as climbing to moderate heights (up to around three metres) without the use of ropes or harnesses. The great variety of difficulty levels in bouldering gyms (usually marked by different colours) allows patients with different physical fitness levels to easily boulder together in one group without feeling over- or unchallenged. Our newly developed bouldering intervention is a combination of bouldering and psychotherapy and consists of 10 consecutive sessions of 2 hours, taking place in a bouldering gym once a week in the late afternoon. The intervention takes place in a group of about 10 patients who are supervised by two climbing therapists. We have teams of two therapists in each study region, but the compositions of personnel may vary across the different waves of therapy because the therapists are also balancing other commitments. All in all, we engage nine climbing therapists for the BPT groups. All but one of the climbing therapists are either psychotherapists or are in the process of completing their psychotherapy hours (psychotherapists must have a master degree in psychology plus an additional 4200 h of further education as a psychotherapist according to German law; the one exception has an Master of Science in Health Science including sports psychology) and are experienced in bouldering and rock climbing (their climbing ability is rated at a minimum of grade 7 on the UIAA (Union Internationale des Associations d’Alpinisme) scale; one person on each team had received advanced training from the German Alpine Association or the Austrian Institute for therapeutic climbing). Prior to the intervention, all of the therapists are thoroughly trained in bouldering techniques, safety rules, and didactic elements by a professional bouldering instructor and in the implementation of the bouldering therapy manual by two members of the study’s headquarters, both of whom had obtained qualifications in “therapeutic climbing”.

Each of the 10 sessions focuses on a specific psychological topic that we consider to be relevant in the development and maintenance of depression. An overview of the specific subjects covered in the 10 therapeutic sessions is provided in Table 2.

**Table 2:** Overview of the bouldering therapy sessions

Session	Topic
1	Introduction to bouldering and mindfulness
2	Physical feeling and the body’s centre of gravity
3	Healthy handling of limitations
4	Expectations and standards
5	Self-efficacy, achievements, and pride
6	Self-esteem
7	Fear and trust I
8	Fear and trust II
9	Social relationships
10	Problem solving, reflecting on lessons learned, and transferring them to daily life

To ensure a standardised implementation of the BPT in all of the three therapy centres, the therapists follow a BPT manual that includes a fixed schedule for each session. Each session consists of three main parts: introduction (about 20 min.), action phase (about 75 min.), and closure (about 25 min.). The introduction phase takes place in an enclosed room (e.g. the yoga room) and begins with a mindfulness exercise (e.g. breathing meditation) to focus the patients’ attention on the current moment, followed by the presentation of the topic of the session and a brief exchange of patients’ experiences and psychoeducation regarding the specific subject (e.g. function and body signals of anxiety). Afterwards, patients are split into two smaller groups, and from then on, each group is supervised by one therapist. The action phase takes place in the bouldering hall and consists of one to two subject-related bouldering exercises that are supposed to evoke underlying emotions (e.g. anxiety), unveil patients’ characteristic patterns (e.g. avoidance), and - with the support of the therapists - enable patients to engage in new experiences (e.g. exposition: bouldering blindfolded). The remaining time in the action phase is used for free bouldering, meaning that patients work on their individual “projects” while supported by the therapists. The closure phase takes part in the enclosed room where all of the patients are reunited to talk about their experiences during the bouldering exercises and to develop ideas about how to integrate the lessons they learned into their daily lives. Each session ends with a body-related relaxation exercise.

#### Cognitive behavioural therapy (CBT)

The CBT group is also manualised and follows the treatment plan of classical cognitive-behavioural therapy. It combines the psychoeducational parts of Schaub et al. [38] with specific exercises as well as elements of the strategic behavioural therapy (SBT) by Sulz [39] and elements of the short-term concept by Hautzinger & Kischkel [40]. At the beginning and end of each session, mindfulness-based techniques and relaxation methods are integrated. Comparable to the BPT, the CBT intervention consists of 10 consecutive sessions of 2 hours, takingplace once a week in the late afternoon in a group of about 10 patients with teams of two therapists in each study region (all in all, eight different therapists). Comparable to the qualification of the BPT therapists, all CBT therapists are either psychotherapists or psychologists who are completing their psychotherapy hours and are experienced in the treatment of depression. Prior to the intervention, all of the therapists are thoroughly trained in the implementation of the CBT manual by two members of the study’s headquarters. Similar to the BPT intervention, each of the 10 sessions focuses on a specific psychological topic (see Table 3).

**Table 3:** Overview of the cognitive behavioural therapy sessions

Session	Topic
1	Introduction to CBT therapy and psychoeducation
2	Psychoeducation on depression and dysfunctional beliefs
3	Identifying and working on individual dysfunctional beliefs
4	Behavioural activation/ activity scheduling
5	Social relationships I
6	Social relationships II
7	Cognitive techniques I
8	Cognitive techniques II
9	Lessons learned: Transforming dysfunctional beliefs
10	Reflecting on lessons learned and on transferring them to daily life

Therapists in all of the three therapy centres follow a manual including a fixed schedule for each session. Each session consists of three main parts – comparable to the BPT manual: a) introduction – which consists of a mindfulness exercise, a short interactive repetition of the last session, and a review of the home-based exercises; b) main part – in which the specific topic is interactively developed using flipcharts, exercise sheets, and small-group work; and c) closure – where worksheets are handed out, home-based exercises are discussed, and a relaxation exercise (progressive muscle relaxation according to Jacobsen) [41] is carried out.

#### Exercise program (EP)

The home-based Exercise Program consists of a 20-min physical training program, which addresses the same muscles used in bouldering or climbing. At the beginning of the intervention, all participants receive a training manual (including instructions and explanations of all of the exercises), a training DVD, training material (e.g. a multifunctional latex band and training rings to enhance finger and underarm power), as well as psychoeducational material explaining the link between physical activity and mood. Participants are instructed to engage in the training program on their own at home approximately three times a week for the study period of 10 weeks. They receive reminders via e-mail to motivate them to keep on exercising. At the midpoint of the intervention (5 weeks), they are sent additional motivational material via postal mail. After 10 weeks, participants are asked how often they conducted the exercises. After the second measurement point, former EP participants are offered the opportunity to participate in a subsequent 10-week bouldering group, which follows the same treatment plan as the BPT group.

### Data collection

The collection of the outcome data is being conducted via CATIs with the patients. The data are being collected before the beginning of the intervention (t0), at the end of the 10-week intervention period (t1), and 3 months (t2), 6 months (t3), and 12 months (t4) after the end of the intervention period. To prepare patients for the interview, they are sent the questionnaire a few days before the interview takes place. The CATI interviewers are clinical psychology students who are trained at the study’s headquarters.

### Measures

The measures employed at the different measurement points are shown in Fig. 1.

#### Primary outcome measures

*Montgomery-Asberg Depression Rating Scale (MADRS)* [42]. The MADRS is one of the most commonly used rating scales for assessing the core symptoms of depression [43]. It is conducted as a semi-structured clinician-rated interview consisting of 10 items: apparent sadness, reported sadness, inner tension, reduced sleep, reduced appetite, concentration difficulties, lassitude, inability to feel, pessimistic thoughts, and suicidal thoughts. The items ask patients about their experiences during the last week. Each item is rated on a seven-point scale from zero to six with higher scores indicating greater severity of symptoms. A score greater than 31 on the MADRS indicates severe depression, whereas a score of 10 or below indicates remission [44, 45]. The interrater reliability is high and it is sensitive to change [42]. Originally, the scale was published without wording suggestions for clinicians to help them collect the information required to rate the items. For this reason, we used the *structured interview guide for the Montgomery-Asberg Depression Rating Scale (SIGMA)* [43]. The SIGMA is a structured interview guide that offers a selection of different questions for each of the 10 items with good to excellent interrater reliabilities [43].

*The 9-Item Patient Health Questionnaire (PHQ-9)* [46, 47]. The PHQ-9 is a short self-assessment tool often used in primary care settings to screen for depression [48]. Its nine items cover the nine DSM-IV criteria and are rated on a four-point scale ranging from zero (“not at all”) to three (“nearly every day”). The items ask patients about their experiences during the last 2 weeks. The total sum score suggests varying levels of depression. Scores from 0 to 4 indicate minimal depression, scores from 5 to 9 mild depression, scores from 10 to 14 moderate depression, scores from 15 to 19 moderately severe depression, and scores from 20 to 27 severe depression [37, 46]. In the German validation study of the PHQ-9 a cut-off point of 8 had the best relation of sensitivity and specifity [37]. The PHQ-9 is well validated and sensitive to change [49].

#### Secondary outcome measures

*EuroQol Five Dimension Questionnaire (EQ-5D)* visual analogue scale *(VAS)* [50]. The EQ-5D assesses health-related quality of life. For the purpose of this study, we included the VAS to ask patients to rate how good or bad their health-related quality of life (HRQoL) is today. The scale is numbered from zero (“worst HRQoL”) to 100 (“best HRQoL”). It was found to be practicable and useful for the application in the general population [51].

*Questionnaire on Resources and Self-Management Skills (Fragebogen zur Erfassung von Ressourcen und Selbstmanagementfähigkeiten, FERUS)* [52]*.* The FERUS assesses individuals’ health-related resources and manageability. It consists of seven scales, but we included only the *coping* subscale with 12 items. Items are rated on a five-point Likert scale that ranges from one to five, with higher test scores indicating good coping skills. All scales of the FERUS have high convergent and discriminant validities and the retest reliabilities are good to satisfying [52, 53].

*Body Image Questionnaire (Fragebogen zum Körperbild, FKB-20)* [54]. The FKB-20 assesses body image disturbances and subjective aspects of body experience. It consists of two subscales of 10 items each. For the purpose of this study, we included only the *vital body dynamics* subscale. Each item is rated on a five-point scale ranging from one (“strongly disagree”) to five (“strongly agree”) with higher values indicating a more positive body image. The statistical properties proved to be satisfying [55].

*Generalized Anxiety Disorder 7 (GAD-7)* [47, 56]. The GAD-7 is a brief self-reported questionnaire for assessing generalized anxiety disorder. Patients are asked how often they have felt bothered by each of the seven core symptoms of generalized anxiety disorder during the last 2 weeks. Items can be rated on a four-point scale ranging from zero (“not at all”) to three (“nearly every day”). Scores range from zero to 21, with scores of ≥5, ≥ 10, and ≥ 15 indicating mild, moderate, and severe anxiety symptoms, respectively. The GAD-7 proved to be a reliable and valid instrument [56].

*General Self-Efficacy Scale (GSE)* [57]. The GSE measures optimistic self-beliefs about coping with a variety of difficult demands in life. Its 10 items can be rated on a four-point scale ranging from one (“not at all true”) to four (“exactly true”). The total score ranges from 10 to 40 with higher scores indicating higher self-efficacy. The GSE has good psychometric properties [58].

*Rosenberg Self-Esteem Scale (R-SES)* [53, 59]. The R-SES is a self-report instrument for evaluating global self-worth by measuring both positive and negative feelings about the self. Its 10 items can be answered on a four-point scale ranging from zero (“strongly disagree”) to three (“strongly agree”) with higher values indicating stronger self-esteem. The psychometric properties of the scale are satisfactory [60].

*Symptom-Checklist (SCL-90)*. The SCL-90 is a self-report inventory that is used to examine the global intensity of psychological symptoms and distress experienced during the past 7 days using a five-point Likert-type scale ranging from zero to four. The SCL-90 covers nine symptom dimensions, but for the purposes of this study, we used only the *interpersonal sensitivity* subscale. Ratings are summed for each subscale with higher scores indicating greater severity of symptoms. The SCL-90 is a reliable instrument and has satisfying convergent and discriminant validities [61].

#### Other measures

##### Screening questionnaire

The screening questionnaire is filled out by individuals interested in participating in the study after attending the informational sessions for the purpose of assessing the inclusion and exclusion criteria. It consists of sociodemographic data such as age and gender as well as open questions that reflect the criteria for inclusion and exclusion (e.g. height and weight to calculate BMI, current therapeutic treatment, physical limitations, psychiatric comorbidities, and depressive symptoms as assessed with the PHQ-9).

The following data are being assessed via the CATIs in addition to the questionnaires presented above*:*

*Sociodemographic data* (e.g. family status, level of education).

Data regarding *current occupation* (e.g. ability to work, current sick-leave status).

*Medical history regarding depression* (onset, duration, and progression of depression).

*Current therapeutic treatment* (medical, psychological, and drug treatment as well as inpatient treatment).

*Critical life events* during the past 6 months (e.g. death of a relative).

*Physical activity* (frequency and kind of activity beyond activities included in the study).

*Attitude towards physical activity* (positive or negative attitude).

*Attitude towards future group allocation* (EP, CBT, BPT, comfortable, uncomfortable, no preference)

##### Avoidance behaviour

On a self-developed five-point scale ranging from zero (“not at all”) to four (“extremely”), we ask patients whether and how much they avoid certain places, public transportation, special situations, or other.

### Data quality management

All of the therapists and interviewers involved in the study are thoroughly trained for their respective tasks at the study’s headquarters. Therapists and interviewers constantly remain in close contact with the study’s headquarters and discuss all upcoming questions and therapeutic progress in regular appointments. For questions or to eliminate uncertainties, the study headquarters’ staff can be contacted anytime during the entire study period. Treatment adherence is documented in protocol assessment surveys, which are filled out by the therapists after each session (e.g. deviations from the manual, severe adverse events, and other extraordinary events). To obtain evidence of the inter-rater reliability of the SIGMA, 5% of the pre- (t0) and 5% of the post- (t1) intervention data are being collected with the participation of a second person who is observing. The quality of the data is guaranteed by strict data monitoring at the study’s headquarters over the entire period of data collection.

The patient information sheet contains a data protection declaration that is in accordance with European and German data protection laws. Sensitive data collected during the telephone interviews is pseudonymised and stored in password-protected devices. Names and addresses of the participants are recorded separately and password-protected. Patient information sheets and the screening questionnaires are stored in a locked steel cabinet. Only members of the study team have access to the lists of the names and codes of participants. No published material will contain patient identifying information.

### Data analysis

The data analyses will be performed with the “IBM SPSS Statistics 21” software. In order to be able to assess the quality of the randomisation, the baseline data from the intervention and control groups will be examined for statistically significant differences. All data will be checked for plausibility. Patients who drop out of the study but are still available for the telephone interviews are being interviewed subsequently. A missing data evaluation will be carried out, and missing values will be imputed by EM-Imputation [62]. The main outcome criterion is severity of depression assessed by the MADRS. The primary data analytic strategy is “per protocol” for the superiority trial and “intention to treat” for the non-inferiority trial [63]. As a sensitivity analysis, an additional analysis with “intention to treat” for hypothesis 1 and “per protocol” for hypothesis 2 will be performed. The level of statistical significance is set at *p* = 0.05.

#### Hypothesis I: superiority of BPT over EP

The primary outcome variable is the MADRS. To test the first hypothesis, t-tests for independent groups of change scores (pre-post) will be performed between the BPT and EP groups. In addition, multivariate analyses (ANOVAs and multiple linear regression analyses) will be calculated. To control for the effects of confounders, demographic variables as well as other therapeutic treatments patients used outside of the treatments offered in the study (e.g. antidepressive medication, psychotherapy, offers to participate in additional sports) will be included in the regression analyses.

#### Hypothesis 2: non-inferiority of BPT to CBT

The second hypothesis will be tested by conducting a non-inferiority analysis with both measurements for depression, the PHQ-9, and the MADRS. The reason for this is that a predefined equivalence margin for the PHQ-9 already exists in literature, but this is not the case for the MADRS. For the PHQ-9, we will use the established equivalence margin of 1.9 points [36, 64]. For the MADRS, only two predefined equivalence margins can be found in the literature, both established for medical treatment against placebo and not for CBT as in our study [65, 66] .

Historical trials of cognitive behaviour group therapy have yielded effect sizes of Cohen’s d ranging from 0.4 [67] to 0.68 [68] to 1.3 [35]. The non-inferiority margin can be chosen as half of the mean difference between state-of-the-art therapy and the control treatment [63, 69, 70]. Because it is easier to find non-inferiority if the margin is larger, we will calculate it with the two lower margins of 0.2 (0.5*0.4) and 0.34 (0.5*0.68) from the two current meta-analyses. Therefore, we will calculate Cohen’s d / Hedges’ g of the between-group difference (BPT-CBT).

Furthermore, the difference between CBT and the active control group EP will be calculated, and the mean difference will be used as a supplementary non-inferiority margin as proposed by different authors [63, 69, 70], who suggest controlling for one’s own standard therapy by using an additional control group if historical trials for the estimation of the non-inferiority margin are missing. Because our EP group is designed as an active control group with a therapeutically active intervention (physical activity), the margin found in the comparison between CBT and EP is likely to be conservative compared to an inactive control group. Various authors [63, 69, 71] recommend the definition of the margin based on clinical considerations. At an average severity interval of about 10 points (below 10 points remission, 10 to 20 points mild depression, to 31 points moderate depression [44, 45]), a reduction of at least 5 points appears clinically relevant. The margin to be determined should therefore not exceed 5 points.

#### Secondary measurements

Secondary outcome variables such as self-esteem and anxiety will be tested exploratively with t-tests for independent groups.

## Discussion

This is the first study to investigate the effect of a bouldering psychotherapy (BPT) on outpatients’ depressive symptoms compared with evaluated treatment options - namely mere physical activity (superiority analysis) and state-of-the-art cognitive behavioural therapy (CBT, non-inferiority analysis).

The results of the study may contribute to an enlargement of treatment options for outpatients suffering from depression. As bouldering is getting increasingly popular, participation in a bouldering therapy might be more socially accepted and thus may represent a lower threshold-offering than participation in classical psychotherapeutic approaches.

Participants of the study will probably benefit, as they receive additional therapy without incurring additional costs or wait times. All study arms were demonstrated to be effective in the treatment of depression prior to this study. Participants are free to start any additional treatments offered by the German health care system while participating in this study without suffering any disadvantages from study personnel, and patients’ individual ongoing treatment plans are not altered by their participation in this study.

Participants in the BPT group have a slight risk of injuries, but this risk does not exceed the risks of other physical activities. A study on injuries in sport-climbing (which has higher risks of severe injuries than bouldering) with about 2000 climbers revealed an average number of 0.2 injuries per 1000 h of climbing [72]. In our study, participants boulder for a maximum of 10*2 h in total. In our pilot study [33, 34], no SAEs in the BPT occurred in around 2400 bouldering hours. Thus, no serious injuries are expected. Nevertheless, SAEs such as strains, ligament ruptures, or fractures as well as suicidal attempts or actual suicides are documented by the therapists for each group and interim analyses of SAEs will be performed on a regular basis. If any analyses show a cumulation of SAEs in the BPT, being significantly higher than in the CBT and the EP group, the DMSB will be consulted.. If a causal connection with the intervention can be plausibly established, the BPT will be terminated and all patients will be offered CBT instead. In addition, accident insurance is being included in the trial participation for all patients. In case of physical injuries during the BPT sessions, the affected person will be stopped from bouldering and sent to an appropriate specialist for medical clarification. Suicidal thoughts between two sessions should be shortly debriefed with the participant prior to the beginning of the session and a suicidal assessment should be carried out to decide whether the participant can distance him- or herself from suicidal tendencies. In case of a suicidal attempt, the participant will be hospitalized and excluded from the study.

All participants are asked to sign an anti-suicide contract for the duration of the study in the screening questionnaire. Interested persons who are not willing or able to complete the anti-suicide contract are not included in the study but are redirected to other treatment options with higher intensity (e.g. inpatient treatment). During the therapies, all therapists follow good clinical practice in monitoring for suicide risk, and suicide risk management plans in cases of acute risk are established by the study centre.

Strengths of the study design are the randomisation, the three study arms with evaluated treatment options as active control groups and the longitudinal character of the study with a long follow-up period of 1 year after the end of treatment. The use of manualised treatments in a naturalistic setting with recruitment of patients in different regions throughout Germany (with a balanced ratio of urban and rural regions) contributes to a high internal and external validity of the study. In order to be able to replicate and implement the treatment, it is planned to publish the manual of the BPT if results of the study show promise.

As our treatment is a non-drug therapy, it must be given open-label and participants and therapists cannot be blinded. In order to reach high objectivity of the results, CATI interviewers, who assess the outcome data are blinded to group allocation of the participants.

We decided to allow patients to receive additional care and defined only few in- and exclusion criteria. This was due to ethical reasons, as we did not want to disrupt ongoing treatment plans as well as to our approach of a naturalistic design. This however has the disadvantage of clinical heterogeneity of the sample, which may dilute the intervention effects.

## Abbreviations

ADHD: Attention deficit hyperactivity disorder
BMI: Body mass index
BPT: Bouldering psychotherapy
CATI: Computer-assisted telephone interview
CBT: Cognitive behavioural therapy
EM-Imputation: Imputation using an expectation maximization algorithm
EP: Exercise program
EQ-5D: EuroQol Five Dimension Questionnaire
FERUS: Questionnaire on Resources and Self-Management Skills
FKB-20: Body Image Questionnaire
GAD-7: Generalized Anxiety Disorder 7
GSE: General Self-Efficacy Scale
HRQoL: Health-related quality of life
MADRS: Montgomery-Asberg Depression Rating Scale
PHQ-9: 9-Item Patient Health Questionnaire
R-SES: Rosenberg Self-Esteem Scale
SAE: Severe adverse events
SCL-90: Symptom-Checklist
SD: Standard Deviation
SIGMA: Structured interview guide for the Montgomery-Asberg Depression Rating Scale
UIAA scale: Scale of difficulty of the Union Internationale des Associations d’Alpinisme (International Union of the Alpine Associations)
VAS: Visual analogue scale
